# Assessment of Environmental Impact on Glass-Fiber-Reinforced Polymer Pipes Mechanical and Thermal Properties

**DOI:** 10.3390/polym16131779

**Published:** 2024-06-24

**Authors:** Cătălina Călin, Alin Diniță, Gheorghe Brănoiu, Daniela Roxana Popovici, Maria Tănase, Elena-Emilia Sirbu, Alexandra-Ileana Portoacă, Sonia Mihai

**Affiliations:** 1 Chemistry Department, Petroleum-Gas University of Ploiești, 100680 Ploiesti, Romania; catalina.calin@upg-ploiesti.ro (C.C.); elena.oprescu@upg-ploiesti.ro (E.-E.S.); smihai@upg-ploiesti.ro (S.M.); 2 Mechanical Engineering Department, Petroleum-Gas University of Ploiești, 100680 Ploiesti, Romania; adinita@upg-ploiesti.ro (A.D.); alexandra.portoaca@upg-ploiesti.ro (A.-I.P.); 3 Petroleum Geology and Reservoir Engineering Department, Petroleum-Gas University of Ploiești, 100680 Ploiesti, Romania; gheorghe.branoiu@upg-ploiesti.ro

**Keywords:** GFRP, environmental effects, mechanical properties, tensile strength, flexural strength structural properties, XRD, TGA, FTIR

## Abstract

Glass-fiber-reinforced polymer (GFRP) composites are widely used due to their high strength-to-weight ratio and corrosion resistance. However, their properties can degrade under different environmental conditions, affecting long-term reliability. This study examines the effects of temperature and chemical environments on GFRP pipes. Specimens were exposed to salt water and alkaline solutions at 20 °C and 50 °C. Diffusion coefficients and tensile and flexural properties were measured. Advanced techniques (TGA, FT-IR, and XRD) showed a 54.73% crystallinity difference between samples at 20 °C/air and 50 °C/salt water. Elevated temperatures and alkaline conditions accelerated degradation, with diffusion coefficients 68.38% higher at 50 °C/salt water compared to at 20 °C/salt water. Flexural strength decreased by 47.65% and tensile strength by 13.89%, at 50 °C/alkaline compared to 20 °C/air. Temperature was identified as the primary factor affecting mechanical performance, while alkaline environments significantly influenced tensile and flexural modulus. These results underscore the importance of considering environmental factors for the durability of GFRP composites.

## 1. Introduction

Glass-fiber-reinforced polymer (GFRP) composites are very used in numerous industries due to their exceptional strength-to-weight ratio, resistance to corrosion, and versatility [1]. These materials find extensive applications in sectors ranging from aerospace and automotive to civil engineering and marine structures [2]. However, the durability and mechanical integrity of GFRP composites under various environmental conditions remain a significant concern. Their exposure to moisture, temperature variations, and chemical environments can lead to the degradation of their material properties, necessitating a thorough understanding and characterization of these effects to predict their service life and ensure their reliability [2,3,4,5].

In our quest to explore the effects of aggressive environments on FRP, we adopted a systematic approach starting with an extensive search of the academic literature. This search was conducted using the Web of Science (WOS) database, focusing on papers containing the keywords FRP, GRP (Glass-Reinforced Polymers), aging, mechanical properties, and environment. By using these specific keywords, we aimed to encompass a broad spectrum of relevant literature and capture studies examining the mechanical behavior of FRP materials under various environmental conditions.

**Figure 1:**
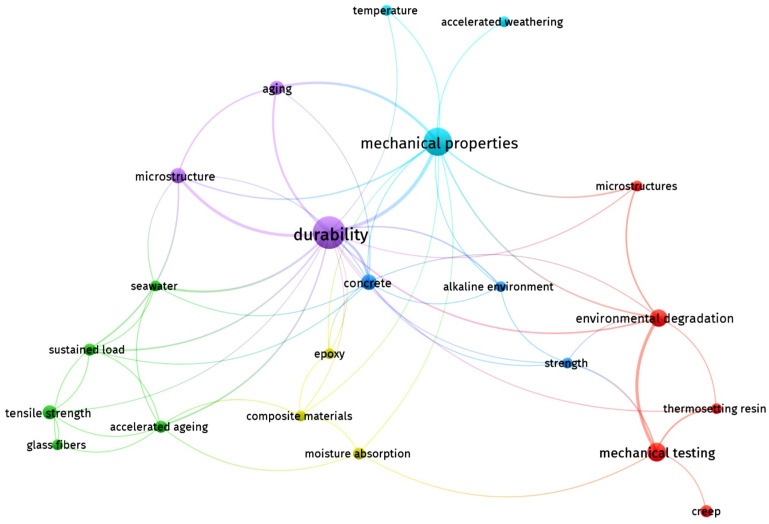
Network visualization of FRP keywords (source: authors, based on analyzed papers).

After compiling the relevant papers, we utilized VOSviewer version 1.6.20, a robust bibliometric software tool, to perform a cluster analysis, as illustrated in Figure 1. This analysis enabled us to visualize and identify clusters within the literature, helping us discern prevalent themes, emerging trends, and potential research gaps.

The network depicted in Figure 1 indicates extensive research on general mechanical properties and environmental effects. However, there is a notable lack of specific studies on the combined mechanical and thermal behavior of GFRP pipes under various environmental conditions, underscoring a notable research gap and presenting an opportunity to provide innovative insights.

By focusing on the mechanical and thermal behavior of GFRP pipes, this study addresses a specific gap in the current literature. It integrates various environmental factors such as temperature, moisture, and aging processes, providing a comprehensive understanding that is not well-covered in existing research. We have employed advanced methods, including TGA, FTIR, and XRD analysis, combined with mechanical testing (tensile and bending), to achieve this. The study aims to provide valuable data and insights for industries utilizing GFRP pipes, offering practical implications for their use in diverse environmental conditions. This study provides critical data for optimizing the design and durability of GFRP pipes specifically in the oil and gas industry. By understanding how these pipes perform under various environmental conditions, such as temperature fluctuations and moisture exposure, the findings enable more reliable and cost-effective pipeline solutions. This can lead to enhanced safety, reduced maintenance costs, and extended service life for GFRP pipes used in oil and gas extraction, transportation, and processing facilities.

Hydrothermal aging, which involves the exposure of composites to elevated temperatures and moisture, is a critical factor influencing the long-term performance of GFRP composites. The penetration of water molecules into the polymer matrix and along the fiber–matrix interface can lead to plasticization, hydrolysis, and other degradation mechanisms that impair mechanical properties such as tensile strength, flexural modulus, and compressive behavior [2,3,6,7,8]. Studies [3,6,9,10] have shown that moisture absorption can significantly reduce the tensile and flexural properties of conditioned specimens. Moreover, hydrothermal aging accelerates these degradation processes, making it essential to investigate the mechanical behavior under such conditions to extend the application spectrum of GFRP composites.

The importance of understanding the impact of hydrothermal aging on GFRP composites is highlighted in recent research. For instance, Sepetcioglu et al. [9] examined the effect of hydrothermal aging on graphene nanoplatelets (GnPs)-reinforced basalt fiber epoxy composite pipes. The study revealed that while water absorption led to a notable decline in mechanical properties, the presence of GnPs mitigated some adverse effects, underscoring the potential of nanoreinforcements in enhancing the durability of composite materials. Similarly, Muralidharan et al. [10] focused on hybrid composites of carbon/glass and Kevlar/glass fibers, demonstrating that maximum moisture absorption and corresponding tensile strength reduction occurred at higher temperatures and prolonged exposure times. The findings emphasized the critical role of fiber hybridization and environmental exposure in determining the service life of composite materials. Study [3] investigates the dynamic mechanical properties of glass-fiber-reinforced polyester composites after immersion in sulfuric acid at different temperatures. The findings indicate that higher temperatures increase water absorption and deteriorate the fiber–matrix interface, leading to significant property degradation.

The reviewed literature provides valuable insights into the durability and degradation behavior of fiber-reinforced polymer (FRP) composites under various environmental conditions. One significant area of focus is the impact of environmental exposure and degradation mechanisms on FRP materials. Silva et al. [11] examined the degradation of GFRP laminates under accelerated aging conditions, revealing challenges in predicting long-term degradation, especially in salt fog exposure. Additionally, Liao et al. [12] delved into the degradation behavior of E-glass-fiber-reinforced epoxy resin composite pipes under accelerated thermal and hydrothermal aging, emphasizing the severe impact of hydrothermal conditions on mechanical properties. Furthermore, Mahmoud and Tantawi [13] investigated the effects of strong acids on glass/polyester GRP pipes, highlighting the detrimental effects of acidic environments on mechanical properties. Paper [5] evaluates the tensile properties of glass–polyester pipes after exposure to various acidic and alkaline solutions. The results obtained from testing alkaline solutions suggest a notable decrease in tensile properties, particularly as alkalinity levels rise. Conversely, treatment with acids strengthened the composite material, as was evident in the increased tensile properties such as the modulus of elasticity and the tensile strength. Article [13] examines the effects of strong acids on the mechanical properties of glass/polyester GRP pipes at room and elevated temperatures. The study finds significant changes in weight, impact resistance, flexural strength, and hardness after exposure to acids, highlighting the detrimental effects of acidic environments. Among the acids studied, sulfuric acid demonstrates the most severe impact on strength. X-ray diffraction analysis reveals that the constituents of the GRP are affected by the acid exposure.

Another key aspect explored in the literature is the assessment of durability and the development of prediction models. Calabrese and Fiore [14] evaluated the predictive capabilities of a theoretical model on the failure load of pinned hybrid composites aged in a salt-fog environment, demonstrating reliability with a low average error. Tu et al. [15] focused on the durability of GFRP rebars in an alkaline environment, proposing a method for real-time durability prediction based on the degradation of the elastic modulus. Miyano et al. The authors of [16] proposed an accelerated testing approach based on the time–temperature superposition principle for predicting the flexural fatigue life of carbon fiber-reinforced polymer (CFRP) and glass fiber-reinforced polymer (GFRP) laminates. Their work highlighted the dependency of flexural fatigue strength on time and temperature, offering a framework for estimating the long-term performance of these materials in marine environments. Sunny et al. [17] utilized the Arrhenius model to predict the service life of E-glass fibers immersed in water at elevated temperatures. The study employed scanning electron microscopy and Fourier transform infrared spectroscopy to evaluate the morphological changes and elucidate the mechanisms of strength loss. Such research underscores the importance of understanding the interaction between moisture and glass fibers to forecast the longevity of composite materials in service applications. Nakayama et al. The authors of [18] presented a method for predicting the lifespan of FRP under sulfuric acid exposure, providing valuable insights into corrosion resistance and the effectiveness of immersion tests for evaluating long-term durability.

The impact of elevated temperatures on FRP materials was also investigated. Mazzuca et al. [19] studied the effects of elevated temperatures on the mechanical properties of GFRP laminates produced by vacuum infusion, confirming significant reductions in mechanical properties, especially those dependent on the polymer matrix.

In conclusion, the hydrothermal aging of FRP composites significantly impacts their mechanical properties and service life. Comprehensive studies and accelerated testing methodologies are essential to understand the degradation mechanisms and predict the long-term performance of these materials. The insights gained with this research will facilitate the development of more durable composite materials, thereby extending their application in various industries and enhancing their reliability in adverse environmental conditions.

## 2. Materials and Methods

### 2.1. Materials and Conditions of Immersion

For the tested samples, a Glass-Fiber-Reinforced Epoxy (GRE) pipe with a 105.2 mm inside diameter and 5.5 mm wall thickness was used.

**Figure 2:**
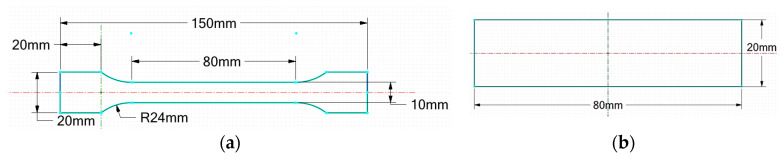
Dimensional characteristics of (**a**) tensile samples and (**b**) bending samples.

**Figure 3:**
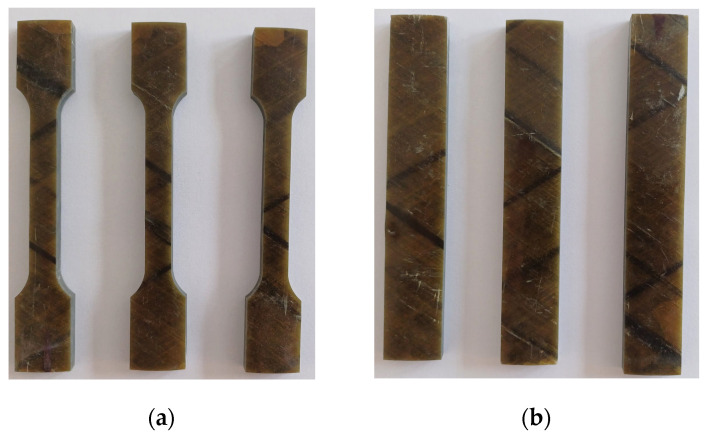
Group of specimens used for performed analyses: (**a**) tensile samples; (**b**) bending samples.

The samples were cut from the pipes using the water jet cutting process with the WUXI YCWJ-380-X1520 (YC WATERJET, Wuxi, China) cutting machine model from the Machining Laboratory of the Mechanical Engineering Department within the Faculty of Mechanical and Electrical Engineering, according to the standard dimensions indicated in [20] (for tensile test) and [21] (for the bending test), as seen in Figure 2. For each case of exposure, three specimens were prepared (see Figure 3).

The solutions were chosen based on significant variations in their pH levels. The pH values of the solutions used were measured using the MULTI 9630 pH meter. The alkaline solution was made according to CSA S806 [22], comprising 118.5 g of Ca(OH)_2_, 0.9 g of NaOH, and 4.2 g of KOH per liter of water, with a measured pH of 13.05. The other environment was 3.5% NaCl (sodium chloride) solution with a measured pH of 7.05.

### 2.2. Design of Experiment

The Design of Experiment (DoE) and Analysis of Variance (ANOVA) techniques involve arrays to organize the factors affecting the behavior of the GFRP material and the levels at which they should be set. A full factorial design with six experimental conditions was established to investigate the effect of the factors’ (levels’) temperatures (20 °C, 50 °C) and environment (air, salt water, and alkaline solution). The testing conditions are presented in Table 1.

### 2.3. Accelerated Ageing Tests of GFRP Pipes

The GFRP specimens were immersed in closed glass recipients containing either salt water or an alkaline solution. Two recipients were placed in an oven to maintain a temperature of 50 °C, and two recipients were put at room temperature (approximatively 20 °C).

The mass gain due to moisture absorption during immersion was evaluated by periodically inspecting the samples, drying their surfaces, and weighing them on a scale with a resolution of 0.01 g.

The calculation of liquid absorbed by the aged samples was performed in accordance to the standard ASTM D5229 [23].

The mass gain percentage was determined using the following equation:

(1) $$M_{t}(\%)=\frac{W_{t}-W_{0}}{W_{0}}\cdot 100$$

where *W_t_* is the weight of the sample at time t and *W_0_* is the initial weight of the sample.

The diffusion coefficient *D* was calculated, using Fick’s law and considering the weight and time taken for the moisture absorption and dimension of both composite samples [6]:

(2) $$D=\pi {\left(\frac{h}{4M_{\infty }}\right)}^{2}\cdot {\left(\frac{M_{2}-M_{1}}{\sqrt{t_{2}}-\sqrt{t_{1}}}\right)}^{2}$$

*h*—the thickness of the composite sample (mm); *M*_∞_—the maximum moisture absorption of the composite (g); and *M*_1_ and *M*_2_ = moisture absorption of the composite sample (g) at time *t*_1_ and *t*_2_ (s), respectively.

After 1080 h (45 days) of immersion, the samples were removed from the solutions and tested for their mechanical and structural properties to investigate the influence of ageing conditions.

### 2.4. Thermogravimetric Analysis (TGA)

Thermogravimetric analysis (TGA) was performed using the TGA 2 STAR SYSTEM (Mettler-Toledo, Columbus, OH, USA) apparatus with a heating rate of 10 °C/min in the range of 25 to 800 °C under 10 mL/min nitrogen.

### 2.5. Fourier Transform Infrared Spectrometer (FTIR)

The functional groups of the samples were evidenced using ATR-FTIR spectroscopy, over the scan range of 4000–400 cm^−1^ using a FTIR Spectrometer Shimadzu IR TRACER-100 (Kyoto, Japan).

### 2.6. X-ray Diffraction (XRD)

In the study with a D8 Advance diffractometer (Bruker-AXS, Karlsruhe, Germany) with θ-θ configuration and Bragg–Brentano geometry with Cu-Kα radiation (λ = 1.54 Å), the samples studied were measured in the range (2θ) 5–70°. The operating conditions of the equipment in XRD Commander were 40 kV and 40 mA, and the scanning conditions were step 0.1° and scan speed 0.1°/5 s. For the qualitative interpretations, DIFFRAC.EVA v14 and the ICDD database were used.

The degree of crystallinity *X_C_* in XRD spectra was calculated using equation [24]:

(3) $$X_{c}=\frac{\sum A_{c}}{\sum A_{c}+A_{am}}$$

where *A_c_* is the fitted areas of the crystal phase, and *A_am_* is the fitted areas of the amorphous phase.

### 2.7. Mechanical Tests

(a) Tensile test

**Figure 4:**
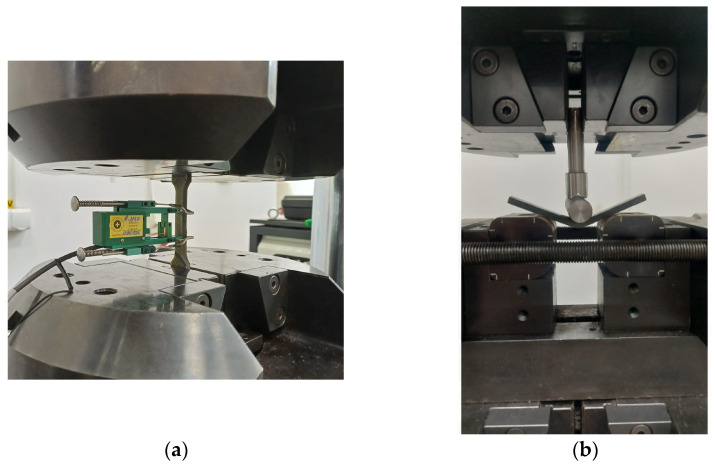
Mechanical testing of GFRP specimens: (**a**) tensile testing; (**b**) 3-point bending testing.

The experimental tests were conducted on the Walter Bai LF300 universal testing machine presented in Figure 4a, with a constant strain rate of 3 mm/min. The tests were conducted at room temperature. The machine was equipped with an extensometer (with a gauge length of 25 mm) to precisely obtain elastic modulus measurements.

(b) Bending test

The flexural specimen was centrally loaded as a simple beam. The flexural test was carried out using the Walter Bai LF300 universal testing machine (Figure 4b), and the testing speed was 5 mm/min, at room temperature (20 °C). The flexural properties (flexural strength σ*_F_*; flexural modulus *E_F;_* and flexural strain at failure ε*_F_*) were determined based on the 3-point bending test according to ASTM D790 [21], with the formulas [25]:

(4) $$\sigma_{F}=\frac{3P_{max}L}{2bh^{2}}$$

(5) $$E_{F}=\frac{mL^{3}}{4bh^{3}}$$

(6) $$\varepsilon_{F}=\frac{6Dh}{L^{2}}$$

where *P_max_* is the maximum bending load (N); *L* is the span length in mm (in this case *L* = 60 mm); *b* and h are the width and the thickness of samples (in mm), respectively; *m* is the slope of the initial straight-line portion recorded in the load-deflection curve; and *D* is the maximum deflection on the beam center (in mm) before failure.

## 3. Results and Discussion

### 3.1. Moisture Absortion

**Figure 5:**
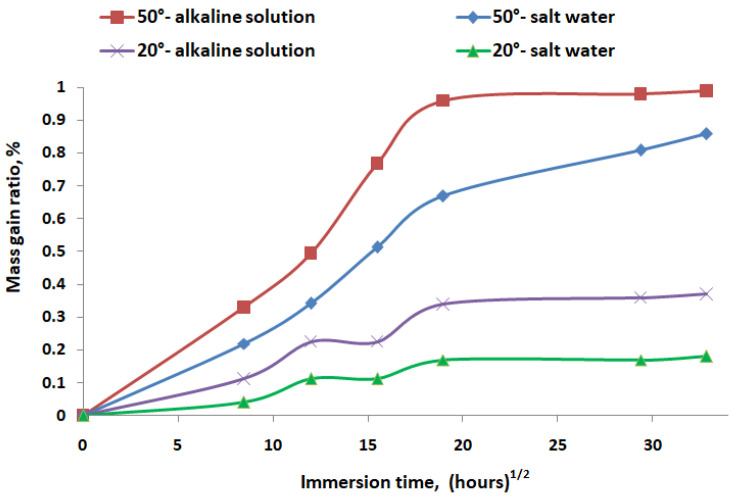
The influence of aging conditions on water absorption of GFRP.

Figure 5 shows the mass gain ratio (%) of GFRP samples immersed in different environmental conditions over time. The square root of time is considered in the analysis of mass gain during immersion because, according to Fickian diffusion theory, the amount of absorbed substance is initially proportional to the square root of time, simplifying the interpretation of diffusion processes [26].

Elevated temperatures (50 °C) significantly increase the mass gain in GFRP pipes, indicating higher absorption or reaction rates with both alkaline and salt solutions. Alkaline solutions cause more mass gain compared to salt water at both temperatures, suggesting that the chemical nature of the alkaline solution more aggressively interacts with or permeates the GFRP material. The combination of the high temperature and the alkaline environment is the most aggressive condition for GFRP pipes, leading to the highest mass gain.

**Figure 6:**
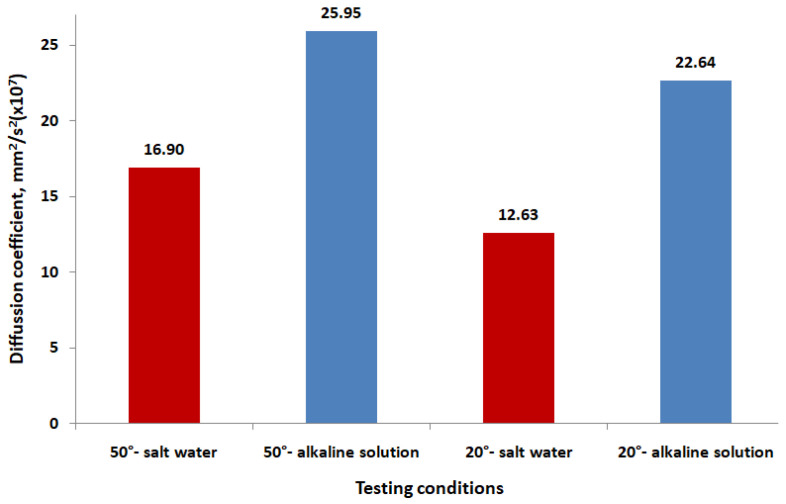
The diffusion coefficient for different testing environments.

The variation in the diffusion coefficient (Figure 6) reveals that higher temperatures (50 °C) generally result in higher diffusion coefficients, indicating that the diffusion process is more pronounced at elevated temperatures, and alkaline solutions consistently show higher diffusion coefficients compared to salt water, both at 20 °C and 50 °C, meaning that the alkaline environment promotes more diffusion into the GFRP material.

### 3.2. Mechanical Properties

#### 3.2.1. Tensile Results

**Figure 7:**
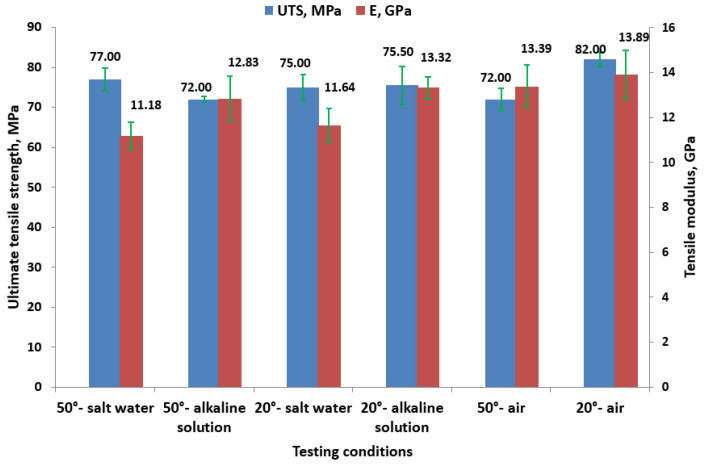
Tensile properties for different testing conditions.

The chart from Figure 7 displays the ultimate tensile strength (UTS) in the MPa and the tensile modulus (E) in the GPa of the samples under various aging conditions.

Higher temperatures (50 °C) tend to reduce the ultimate tensile strength (UTS) compared to lower temperatures (20 °C). The tensile modulus (E) is generally higher at lower temperatures (20 °C), indicating stiffer material behavior under these conditions. In salt water, at 50 °C, the UTS is 77 MPa, and the tensile modulus is 11.18 GPa, while at 20 °C, the UTS slightly decreases to 75 MPa, but the tensile modulus increases to 11.64 GPa. For the samples immersed in alkaline solution, at 50 °C, the UTS drops to 72 MPa with a higher tensile modulus of 12.83 GPa. At 20 °C, the UTS slightly improves to 75.50 MPa with an even higher tensile modulus of 13.32 GPa. These results align with the findings in paper [27], which demonstrate that environmental changes from tap water to seawater or alkaline solutions significantly impact tensile strength. Notably, both studies indicate that alkaline environments exert the most substantial effect on reducing tensile strength.

#### 3.2.2. Flexural Results

**Figure 8:**
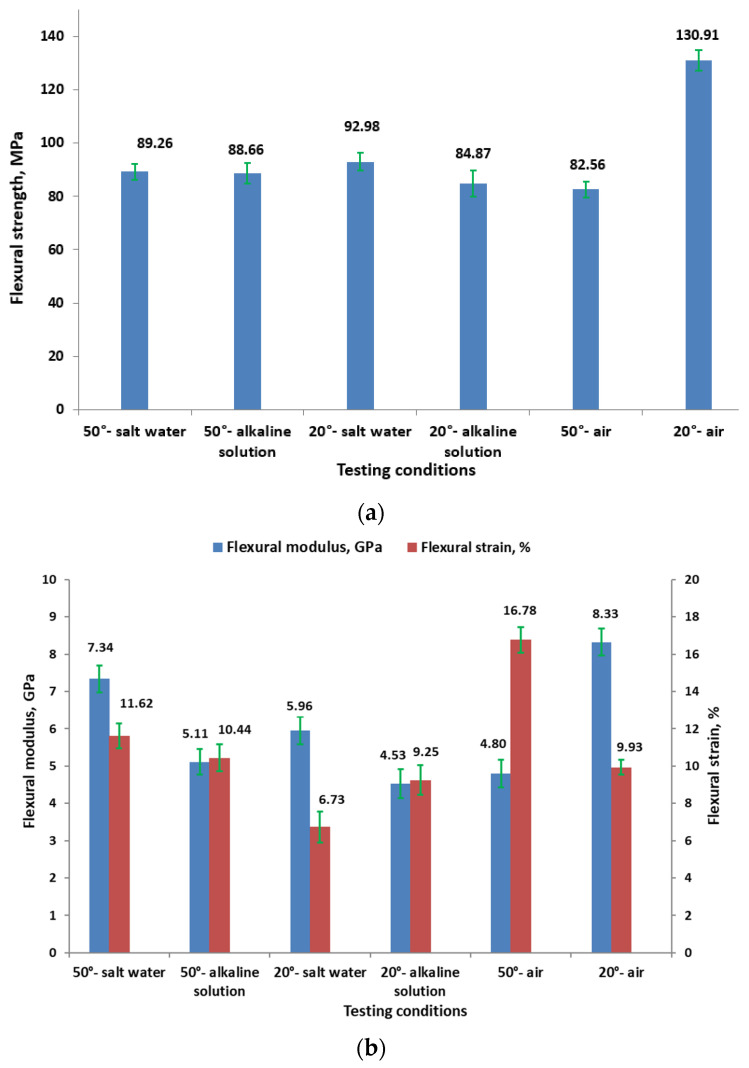
Flexural properties for different testing conditions: (**a**) flexural strength; (**b**) flexural modulus and flexural strain.

It can be observed from Figure 8 that lower-temperature (20 °C) conditions tend to show higher flexural strengths compared to higher-temperature (50 °C) conditions. Salt water at 20 °C also shows relatively high flexural strength (92.98 MPa). Alkaline solution reduces the flexural strength more than salt water at both temperatures. The lowest flexural strength is observed in air at 50 °C (82.56 MPa).

For samples immersed in salt water, at 50 °C, the flexural modulus is higher (7.34 GPa) compared to other 50 °C conditions, but with moderate strain (11.62%). At 20 °C, the modulus drops to 5.96 GPa, with the strain decreasing significantly to 6.73%. For samples immersed in alkaline solution, at 50 °C, the flexural modulus is 5.11 GPa with a strain of 10.44%, while at 20 °C, the modulus further decreases to 4.53 GPa, but the strain increases slightly to 9.25%. To the authors’ knowledge, there are no published studies that specifically analyze the influence of salt or alkaline solutions on the flexural properties of GFRP composites.

### 3.3. TGA Analysis

**Figure 9:**
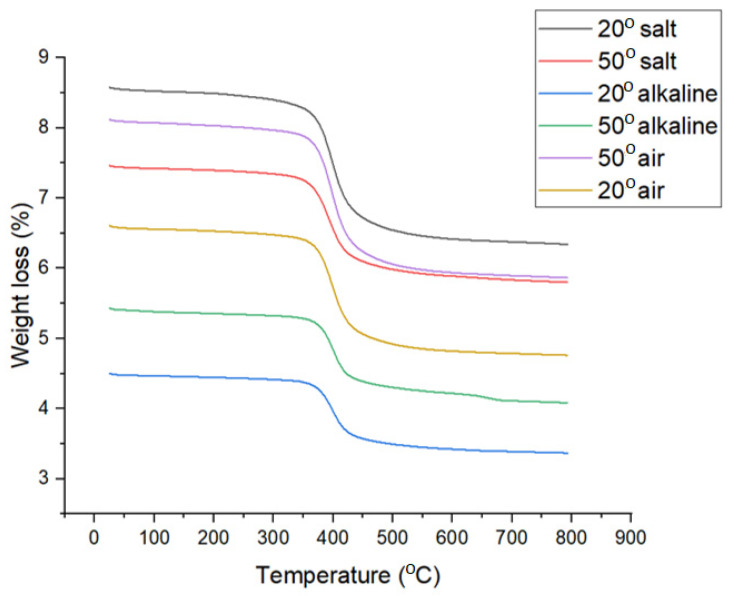
Thermogravimetric analysis of the investigated GFRP samples.

**Figure 10:**
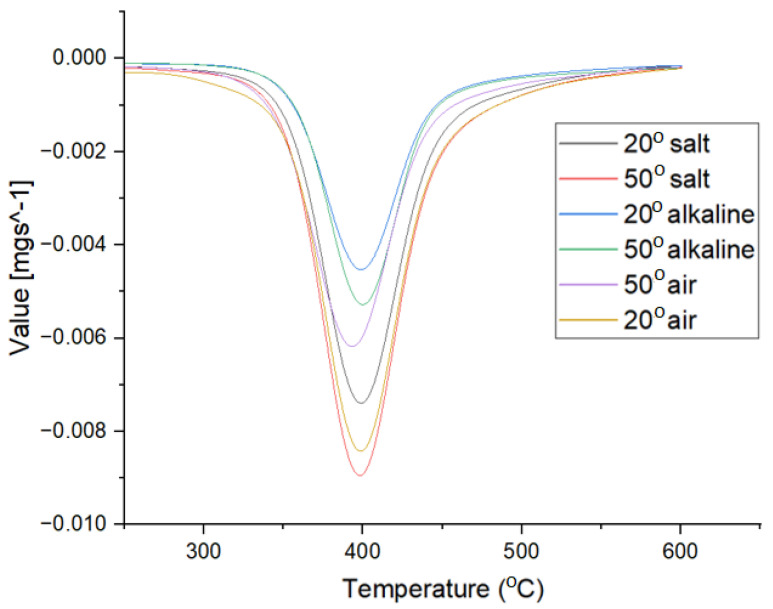
The first derivative of the TGA curves.

**Figure 11:**
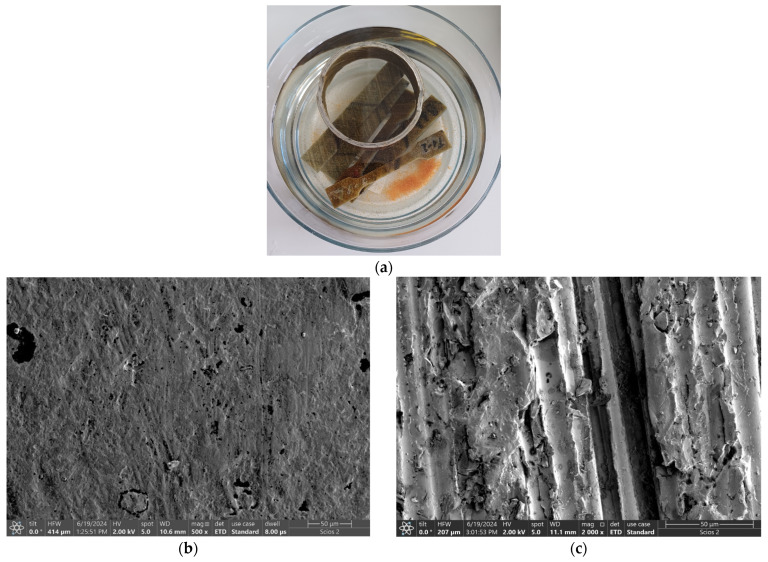
The effect of salt water at 50 °C: (**a**) (orange–brown deposits observed); (**b**) SEM image for reference sample; and (**c**) SEM image for sample immersed in salt water at 50 °C.

The thermogravimetric analyses of the samples are presented in Figure 9. As can be seen, all the samples have the same degradation behavior in three steps. The first stage with a small mass loss between 0.8 and 1.15% was recorded in the range 25–200 °C and is attributed to the loss of humidity and absorbed water [28]. The main degradation step, which occurred between 250 and 560 °C, with a maximum of around 398 °C (Figure 10), was caused by the thermal degradation of the epoxy content of the glass fiber-reinforced polymer [29]. The environmental conditions influence the degradation behavior. Compared to the reference samples (identified as 20°C-air), the samples kept in solution had lower mass losses by 3.32 to 5.67%, suggesting the migration of epoxy resins from polymer into solutions. Thus, the 20°C-alkaline and 20°C-salt samples lost 22.02 and 23.39%, respectively, compared to 25.34% of the control sample. The mass losses are slightly higher for the samples at 50 °C, with 4.79% and 5.67% for the 50 °C-alkali and 50 °C-salt samples, respectively. Moreover, for the samples kept at 20 °C, regardless of the type of solutions, there were no changes in the maximum degradation temperature, but for the samples kept at 50 °C, small shifts of this value were observed. The most significant decrease of 5.34 °C was recorded for the 50 °C-salt sample. This shift may have been due to the degradation of glass fiber epoxy polymer caused by the leaching out of silicate and epoxy content in salt solution [30]. In this case, the deposition of a corrosion precipitate after sample immersion at 50 °C in salt solution (see Figure 11) was observed.

In the SEM images, the difference between the reference (unexposed) sample and the one subjected to the corrosive action can be seen by the fact that the surface of the polymer matrix exfoliates. This correlates with the XRD and FTIR spectra from this study.

### 3.4. XRD Analysis

**Figure 12:**
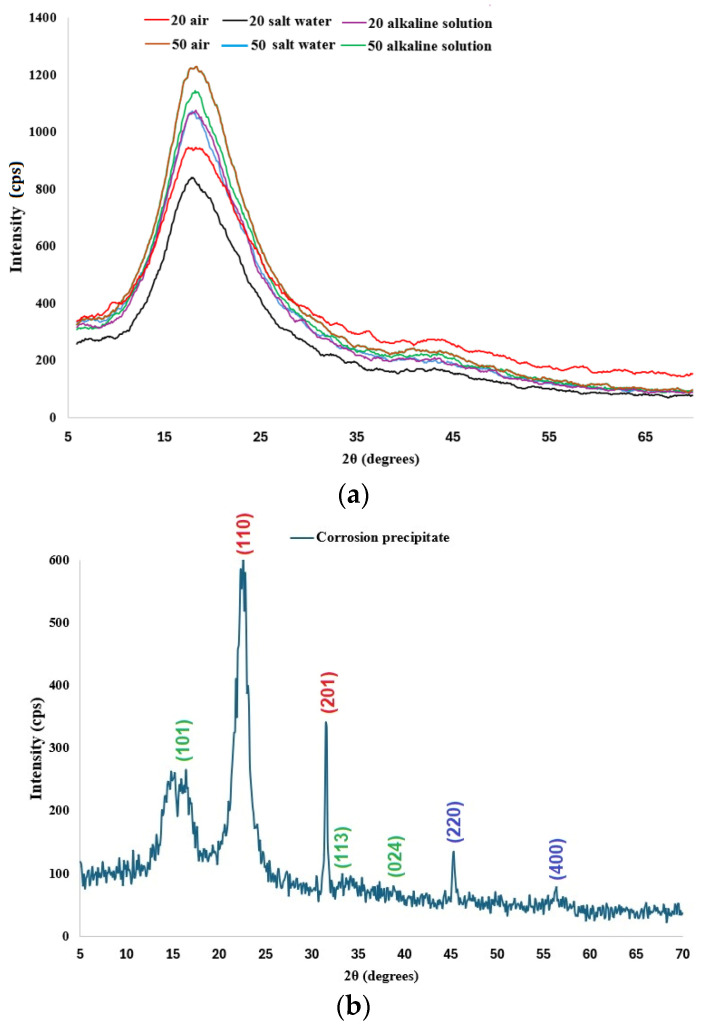
XRD patterns: (**a**) GFRP samples; (**b**) corrosion precipitate.

Although the X-ray spectra show the same trend, a difference can be observed depending on the temperature and the nature of the different solutions (Figure 12).

The XRD spectra of the glass-fiber reinforced polymer (GFRP) show an asymmetric prominent peak in the range 10–30° (2θ degrees), with the maximum peak intensity around 18° (2θ degrees) corresponding to the amorphous character of this type of composites (ICDD-JCPDS database). The major peak at 18° (2θ degree) confirms that the remnant crystalline silica is in the composite. The samples analyzed present similar characteristics with the β-form of GFRP composites, as is indicated in the paper authored by Wang et al. [31].

Qualitatively, in the X-ray spectra of GFRPs, a tendency to increase the peak intensity with the rise of temperature can be observed, suggesting an improvement in the degree of crystallinity (Figure 12a). The absence of other peaks in the X-rays spectra of the composite (GFRP) material can be observed because of the uniform distribution of glass fiber in the matrix phase, indicating the amorphous nature of glass fiber, as is suggested in several previous papers [32,33,34,35,36,37].

XRD qualitative and quantitative investigations were performed on the corrosion precipitate resulting from the exposure of the GFRP sample to the saline environment. For XRD qualitative interpretations, Diffrac. EVA v14 software and PDF-ICDD database were used, and three chemical compounds were identified: iron chlorate hydrate, iron chloride hydroxide, and iron silicon.

The main reflections/peaks observed in the XRD spectra (Figure 12b) expressed in 2θ values are as follows: for iron chlorate hydrate—4.01 (101), 390 (110), and 2.82 (201); iron chloride hydroxide—5.57 (101), 2.83 (113), and 2.32 (024); and iron silicon—2.01 (220) and 1.41 (400), confirming the presence of the three chemical compounds, in good agreement with FTIR analysis.

For XRD quantitative investigations, Topas 4.1 software and the Rietveld refinement method were used. The results of the refinement (wt% Rietveld) of the corrosion precipitate were as follows: iron chlorate hydrate—66.77%; iron chloride hydroxide—26.15%; and iron silicon—7.08%. The GOF (goodness-of-fit) and DW (Durbin–Watson) values were 1.08 and 2.05, respectively, which indicates the good quality of the Rietveld refinement performed [38,39].

**Figure 13:**
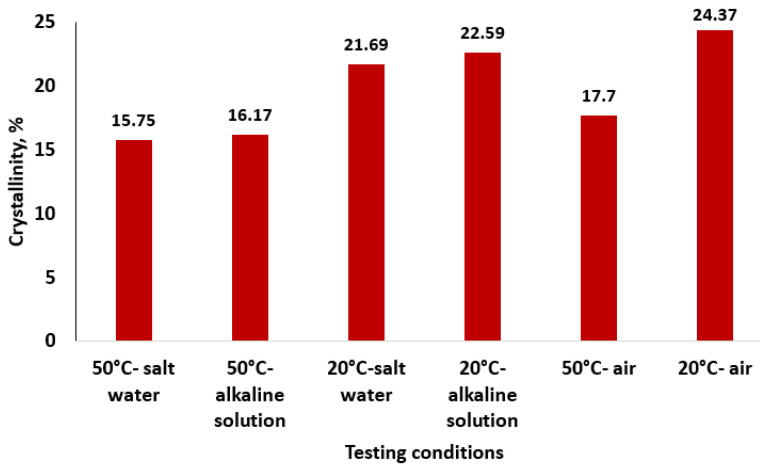
The values of crystallinity for different environments.

The crystallinity results are presented in Table 2 and Figure 13.

The lower values of the degree of crystallinity resulting from XRD are similar to those obtained from different types of GFRP in previous papers [31,32,40,41,42]. The values of the degree of crystallinity calculated from the X-ray spectra show that the degree of crystallinity has the tendency to decrease with the rise of temperature and especially with the salinity of the environment. The decrease in the crystallinity in the samples exposed to different environmental conditions at higher temperatures is explained by the fact that the XRD technique scanned the samples’ surface, which was degraded as a result of the interactions of the GFRP pipes’ surface with different environmental conditions. The XRD data are supported by the FTIR data and by the results of mechanical tests.

### 3.5. FTIR Analysis

**Figure 14:**
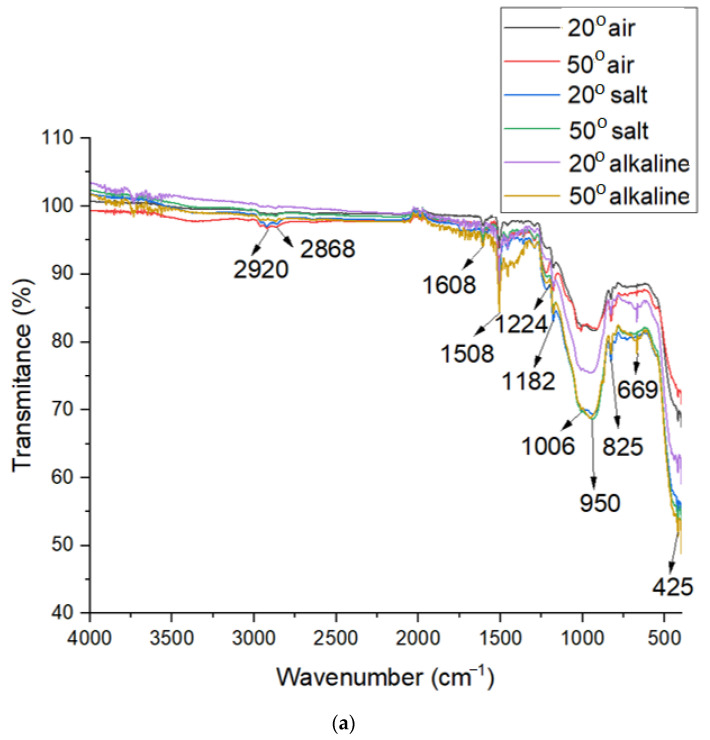

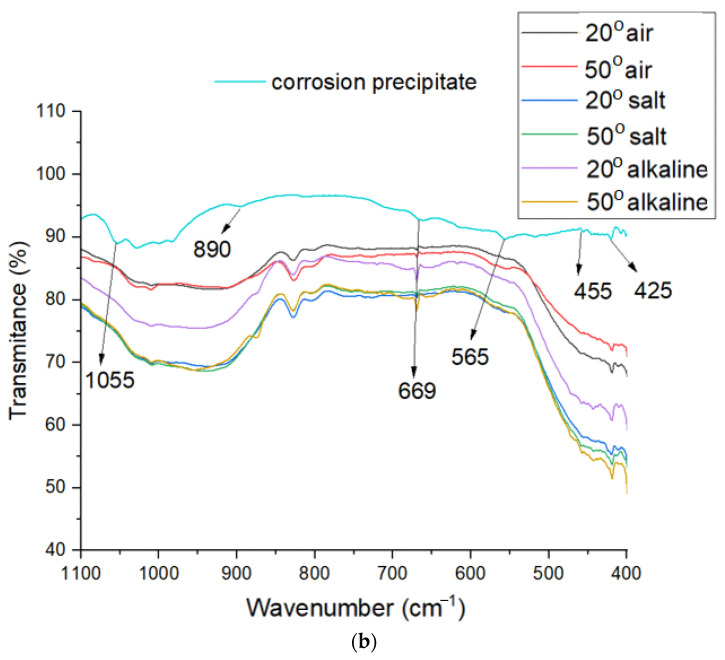
FTIR spectra: (**a**) GFRP samples; (**b**) corrosion precipitate.

As can be seen in Figure 14, all of the samples show peaks specific to the epoxy resin fraction such as the bands at 2920 and 2868 cm^−1^ belonging to the vibrations of the C-H bonds; 1608 and 1508 cm^−1^ attributed to the stretch of C=C bond from the aromatic ring; 1224 cm^−1^ corresponding to the groups ʋ (=C-O) from C_6_H_5_OC; and, finally, 1182 cm^−1^ belonging to the bond δ C-H from the aromatic nucleus [43,44]. The presence of Si-O-Si bonds is indicated by the peaks recorded in the region 1006–425 cm^−1^. An increase in the intensity of the peaks at 1006, 950, and 669 cm^−1,^ attributed to the asymmetric and symmetric vibrations of the Si-O-Si bond [45] and the surface modification of Fe_3_O_4_@SiO_2_ microsphere by silane coupling agent is observed for samples kept both in solutions and at temperature. This behavior is explained by Guo F. et al. [30] by the lower silicate content of the fiber. The Si-OH bending band was observed at 823 cm^−1^. The characteristic absorption peak assigned to the Fe_2_O_3_ band is indicated by the band 425 cm^−1^ [46]. It should be mentioned that the sample exposed to 50 °C in the salt solution produced a precipitate, which, after XRD and FT-IR analysis, supports the above statements regarding the degradation of the samples. According to XRD analysis, the compound contains three structures: iron chlorate hydrate, iron silicon, and iron chloride hydroxide, probably formed due to the hydrolysis of iron and silicon, followed by the reaction with chlorine ions from the salt solution [30]. The absorption bands from the FT-IR spectra at 1055 and 669 cm^−1,^ characteristic of the silicon network and the peaks at 890, 565, and 425 cm^−1^ attributed to Fe-OH, Si-O-Fe and Fe-O bonds, respectively [46], also support the statement regarding the corrosion of the sample.

### 3.6. Statistical Analysis

**Figure 15:**
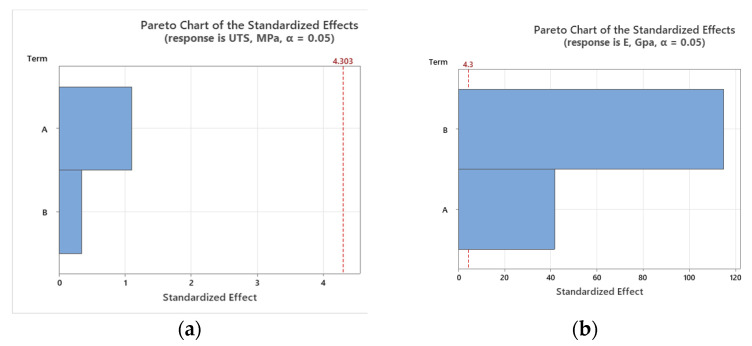
Pareto charts for tensile results: (**a**) tensile strength; (**b**) tensile modulus.

**Figure 16:**
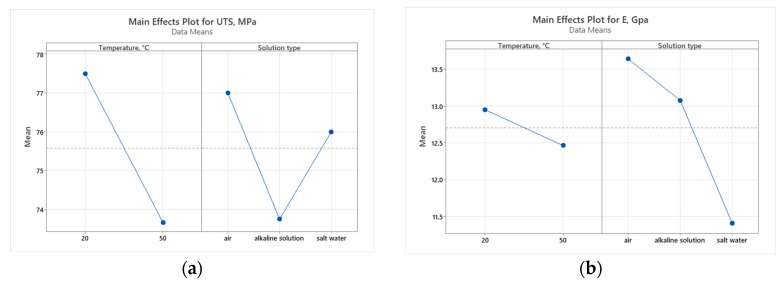
Main effect plots for tensile results: (**a**) tensile strength; (**b**) tensile modulus.

**Figure 17:**
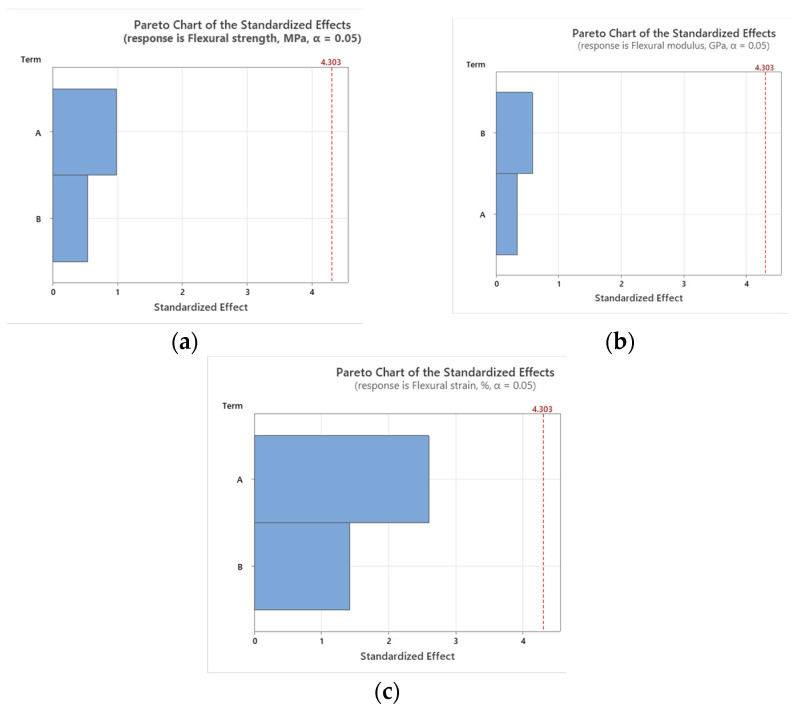
Pareto charts for bending results: (**a**) flexural strength; (**b**) flexural modulus; and (**c**) flexural strain.

**Figure 18:**
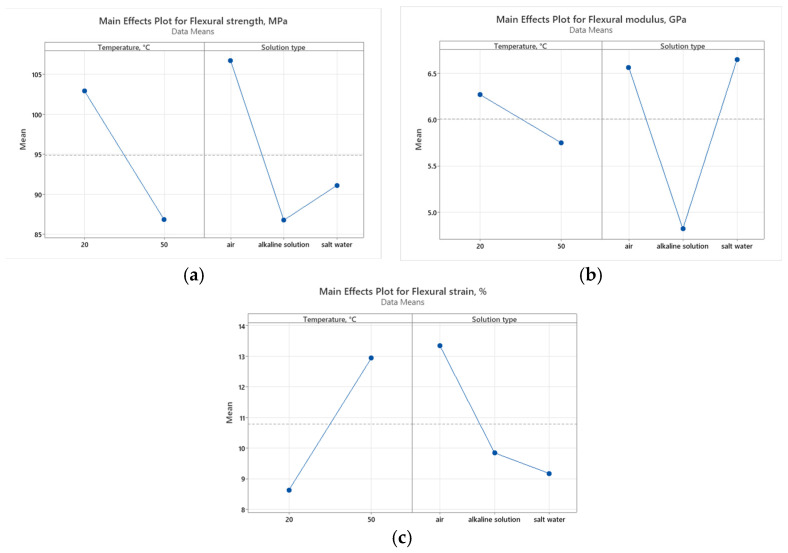
Main effect plots for bending results: (**a**) flexural strength; (**b**) flexural modulus; and (**c**) flexural strain.

To assess the impact of working environment (A—temperature and B—solution type) mechanical properties, the Pareto charts and main effect plots graphical representations are illustrated in Figure 15, Figure 16, Figure 17 and Figure 18.

The large bar for factor A indicates that temperature significantly affects UTS. Similarly, the data from [47] showed that temperature significantly impacts the compressive and tensile strength properties of the GFRP material. Our conclusion also aligns with the findings from paper [27], namely, that temperature significantly impacts the strength properties of GFRP materials (*p*-value < 0.05). The referenced paper [27] also shows that changing the environment from tap water to seawater or alkaline solutions significantly affects tensile strength retention, with alkaline environments having the most substantial impact (97.8%). This highlights the vulnerability of GFRP composites to alkaline conditions. Higher temperatures might lead to a reduction in tensile strength due to material softening or other thermal effects. Although factor B also affects UTS, its impact is less significant than that of temperature. Different solution types might interact with the material in ways that either enhance or degrade its tensile properties, but these effects are secondary to the temperature effects.

The prominent bar for factor B suggests that the type of solution in which the material is immersed has a substantial effect on its elastic modulus. This could be due to chemical interactions that alter the material’s stiffness. While temperature still affects the elastic modulus, its influence is less significant than that of the solution type. This could indicate that the material’s stiffness is more resilient to temperature changes than to chemical exposure.

The plot indicates that increasing the temperature from 20 °C to 50 °C results in a significant decrease in UTS. This suggests that higher temperatures negatively affect the tensile strength of the material, likely due to the thermal softening or degradation of the material structure. The solution type also has a notable impact on UTS. The alkaline solution reduces UTS the most, indicating a possible corrosive effect or weakening of the material. Salt water, while still reducing UTS more than air does, does so to a lesser extent than alkaline solution. The environment in air maintains higher UTS, indicating that the absence of corrosive agents helps preserve the material’s strength.

Temperature has a significant impact on both flexural strength and strain, indicating that higher temperatures likely reduce both the strength and the ductility of the material. This could be due to thermal degradation or changes in the material’s internal structure. The solution type significantly affects the flexural modulus, indicating that chemical interactions are altering the stiffness of the material.

Temperature has a lesser impact on the flexural modulus, suggesting that stiffness is more resilient to temperature changes compared to strength and strain. The solution type also impacts flexural strength and strain, though to a lesser extent than temperature. This implies that while chemical environments affect the material properties, the effect is not as dominant as temperature.

The statistical results demonstrate that temperature is the predominant factor affecting the ultimate tensile strength (UTS), flexural strength, and flexural strain of the material. This finding highlights the critical role of thermal conditions in determining the mechanical performance of GFRP material under various loading conditions. In contrast, the working environment primarily impacts the tensile and flexural modulus. This indicates that the chemical composition and properties of the solution have a significant influence on the stiffness and resistance to deformation of the material. These findings provide new and detailed insights into how different environmental factors uniquely impact the mechanical properties of GFRP materials. Understanding these nuances is essential for optimizing both the thermal and chemical processing conditions, thereby enhancing the overall behavior and durability of GFRP materials in various operational conditions.

## 4. Conclusions

The objective of this study was to investigate the influence of environmental conditions, particularly temperature and chemical exposure, on the mechanical properties and structural behavior of GFRP pipes. Understanding these effects is essential for designing GFRP pipes that can withstand specific environmental challenges, ensuring their long-term durability and performance. The study utilized a combination of advanced characterization techniques to assess the impact of environmental conditions on GFRP pipes, such as XRD (to analyze the crystalline structure and phase composition of the materials), TGA (to measure changes in the physical and chemical properties as a function of temperature), FTIR (to identify the chemical bonds and molecular structure changes due to environmental exposure), and mechanical testing (to measure tensile and bending properties).

The main findings of this study are:

➢ The impact on the diffusion coefficient: High temperatures and alkaline solutions significantly increase the diffusion coefficient in GFRP pipes. This accelerated diffusion can lead to substantial changes in material properties over time, potentially compromising their performance.
➢ The effect on tensile properties: Exposure to higher temperatures (50 °C) and alkaline solutions results in a reduction in UTS, indicating the decreased strength of GFRP pipes under these conditions. Despite this reduction, the tensile modulus remains relatively high, suggesting that the material retains its stiffness, which is crucial for load-bearing applications.
➢ The effect on flexural properties:
  ✓ Higher temperatures and alkaline solutions significantly reduce the flexural strength of GFRP pipes.
  ✓ Higher temperatures (50 °C) reduce the flexural modulus, indicating decreased stiffness, but also increase the flexural strain, suggesting increased flexibility.
  ✓ Alkaline solutions degrade the flexural properties more significantly than salt water.
➢ The primary influencing factors:
  ✓ Temperature is the primary factor affecting UTS, flexural strength, and flexural strain, highlighting the critical role of thermal conditions in determining the mechanical performance of GFRP composites.
  ✓ The solution type, particularly in alkaline environments, mainly influences the tensile and flexural modulus, underscoring the importance of chemical resistance for the long-term performance of GFRP materials.
➢ Behavior in salt water: For samples immersed in salt water at 50 °C, the flexural modulus is higher (7.34 GPa) compared to other 50 °C conditions, with moderate strain (11.62%). This behavior could be explained by the formation of a corrosive precipitate, as shown by XRD and FT-IR analyses.

This study provides valuable insights into the environmental durability of GFRP composites, guiding the design and application of these materials in various industrial settings to ensure their optimal performance and longevity.

## Figures and Tables

**Table 1:** Testing conditions.

Test No.	Temperature, °C	Environment
1	50	salt water
2	50	alkaline solution
3	20	salt water
4	20	alkaline solution
5	50	air
6	20	air

**Table 2:** XRD results regarding crystallinity of GFRP material.

Testing Conditions (Temperature/Immersion Solution)	*A_c_*	*A_am_*	*X_c_*, %
50 °C/salt water	82.017	438.568	15.75
50 °C/alkaline solution	111.738	579.122	16.17
20 °C/salt water	66.004	238.198	21.69
20 °C/alkaline solution	98.663	338.129	22.59
50 °C/air	144.852	673.379	17.70
20 °C/air	106.134	329.407	24.37

## Data Availability

All data are presented within the article.
